# Supplementary material for: Are carbon emissions trading and green financial instruments synergistic? -Comprehensive quantitative research based on content analysis
Source: PLoS One. 2024 Mar 7;19(3):e0298601. doi: 10.1371/journal.pone.0298601 (PMC10919601; doi:10.1371/journal.pone.0298601)
Supplement: S1 Data — (ZIP) [file pone.0298601.s001.zip › Data/地级市Z_F工作报告.zip!/╣π╬≈/╖└│╟╕█╩╨/└┤╫╘╣1⁄2╓┌║┼í╛┬φ┐╦╩2╛▌═°í┐_2005.docx]

各位代表：

现在，我代表防城港市人民政府，向大会作政府工作报告，请予审议，并请政协各位委员和其他列席人员提出意见。

二○○四年工作回顾

过去的一年，在自治区党委、自治区人民政府和市委的正确领导下，在市人大、市政协的监督和支持下，我们坚持以邓小平理论和“三个代表”重要思想为指导，全面贯彻党的十六大和十六届三中、四中全会精神，认真落实科学发展观，解放思想，与时俱进，开拓进取，艰苦奋斗，较好地完成了市三届人大四次会议确定的经济社会发展任务，实现了自治区提出的经济“三突破”目标。全市经济社会协调发展，精神文明和民主法制建设取得新成绩，边境安宁，民族团结，社会和谐稳定，人民安居乐业。

“三突破”目标胜利实现，全市经济快速健康发展。全市生产总值、财政收入和规模以上工业利润全面实现“三突破”目标。初步核算，GDP突破80亿元，达到84.97亿元，增长12.03%；财政收入突破6亿元，达到6.78亿元，增长20.85%；规模以上工业利润突破1亿元，达到1.14亿元。其它主要经济指标保持较快增长。全市港口货物吞吐量完成1929万吨，增长26.9%；其中集装箱8.02万标箱，增长53.1%。全社会固定资产投资30.35亿元，增长54.03%。农业总产值35.93亿元，增长5.7%。工业总产值99.78亿元，增长23.63%；规模以上工业产值65.91亿元，增长29%；规模以上工业增加值11.82亿元，增长27%。外贸进出口总额7.35亿美元，增长51%，其中出口8358万美元，增长14%。边贸成交额32.04亿元，增长22.83%。海关税收23.95亿元，增长69.87%。接待游客156.22万人次，旅游收入5.33亿元，分别增长35.4%和49.7%。金融机构存款总额70.5亿元，比年初增长16.71%；贷款余额40.12亿元，增长7.11%。社会消费品零售总额19.51亿元，增长10.86%；居民消费价格指数103.4%，上升3.4个百分点。产业结构进一步优化，三次产业结构由上年的27.7∶33.3∶39调整为25.9∶37∶37.1。

县域经济发展壮大，农村经济迈上新台阶。县域生产总值初步核算增长10.56%。调减3万亩低产水旱田、咸酸田发展高效经济作物和海水养殖。推广高产高糖甘蔗25万亩。新增良种水果种植9000多亩，建立了一批标准化水果生产示范园，建成全国最大的火龙果种植基地。渔业产值16.89亿元，增长7.06%。畜牧业产值5.06亿元，增长9.72%。完成人工造林12万亩。建成海世通、宝林香精等20多家农产品加工“龙头企业”。县域工业增加值占县域GDP的比重由上年的27.3%提高到28.2%。企沙、江平、那良、峒中、在妙等小城镇规划建设有效推进，企沙被列为全国经济综合开发示范镇。民营经济发展加快，新增个体工商户6598户、私营企业272家。发展劳务经济成效显著，新增农村剩余劳动力转移1.27万人。落实农民增收项目87个，农民人均纯收入2517元，增长7.8%。农村税费试点改革成果得到巩固。农业基础设施建设得到加强，建成渠道硬化、海堤加固、界河整治工程23公里，除险加固水库4座，修复水利水毁工程160宗。改造与建设农村电网台区6个、架设线路38.6公里。

临港工业快速发展，经济效益明显提高。大海粮油、新海油脂生产规模不断扩大，产值分别完成39.97亿元和11亿元，拉动全市规模以上工业经济增长22.7个百分点。上上糖业、昌菱实业生产经营稳步发展。华塑包装、中天药业、鉴声电器、福城钢铁和岳泰饲料等企业相继投产试产。万鑫钢铁、宏源浆纸、中油能源等项目建设加快。启动48家企业产权改革,有8家完成改制。技改投资完成1.7亿元，增长33.6%，创历史新高。产品销售收入增长29.14%，企业亏损面下降4.97个百分点。

重大项目建设步伐加快，发展后劲不断增强。全市33项重大建设项目投资完成19.05亿元。沿海基础设施建设大会战10个项目全面开工建设。防城港20万吨级泊位及配套航道投资完成4.29亿元；13至17号泊位和东湾化工码头投资完成4923万元。东兴至江山一级公路封油通车；沙潭江至企沙一级公路、上思至东兴公路、上思至崇左二级公路防城港市段、企沙工业区路网建设进展顺利。企沙工业区供水管网、沙潭江至企沙供水管网、长歧水利干渠防渗加固扩能、白石牙水库、茅岭和东湾输变电工程及平福水电厂等项目建设加快。防城港发电厂开工准备工作就绪。企沙千万吨级钢铁厂、东湾跨海大桥、中越北仑河二桥及配套公路、大型能源等项目前期工作加快。

城市规划建设管理得到加强，城市化进程稳步推进。完成城市总体规划修编和南北大道两侧城市设计,开展了江山半岛旅游度假区规划工作。批准实施上思县县城总体规划，完善东兴市总体规划。城市基础设施建设得到加强，城市中心区四套班子办公用房和路网建设继续推进。建成市10万吨水厂主管道、市生活垃圾处理厂填埋工程和拥军路西段。市污水处理厂等项目前期工作进展顺利。对兴港大道、滨海路海堤、贵州路、四川路、凯乐园路、出海通道等进行了美化、绿化、亮化。烂尾楼工程处置工作进入全面实施阶段。城市电网配网工程进展顺利。

招商引资力度加大，外向型经济取得新进展。出台招商引资考核奖励办法，建立招商引资新机制。参加中国—东盟博览会，成功举办2004中越边境商品交易会，加强与东盟各国和泛珠三角以及江浙等地区的经贸合作。签订外引内联合同项目59项，合同投资59.59亿元；协议项目87项，协议投资29.7亿元；实际到位资金12.4亿元。新批准成立外商投资企业9个，合同外资1908万美元，实际利用外资625万美元。

就业和社会保障工作不断加强，人民生活水平进一步提高。就业和再就业工作取得新进展。免费提供职业介绍服务和再就业培训3000多人。全市新增城镇就业9056人，完成全年任务113.2%；城镇登记失业率控制在3.5%以内。

社会保障体系逐步健全。养老、失业、工伤、生育参保人数等八项指标超额完成自治区下达的任务，养老保险扩面居全区前列。按时足额发放企业离退休人员基本养老金和失业人员失业保险金，并向1.34万人提供最低生活保障。全市住房公积金实现统一规范化管理，资金归集创历史新高。农村困难群众生活保障得到加强，建成五保村50个。投入300多万元抗旱救灾，把灾害损失降到最低限度。

认真解决事关群众生活的突出问题。财政安排支农资金7000万元。农村税费改革减轻农民负担2500多万元。安排社会保障支出2900多万元。提高个体户税收起征点，使1200多人次享受税收减免政策。清理整顿各类开发区，逐步兑现历年的征地款和拖欠的工程款。农民工工资清欠取得阶段性成果。加强市场价格监管，消费者权益得到保护。教育、医药行业乱收费问题得到有效治理，减轻群众负担400多万元。

扶贫攻坚和“兴边富民”向纵深推进。南屏瑶族乡基础设施建设大会战投入2900多万元，完成项目68个。实施农村人饮工程24宗，解决2.38万人的饮水困难。“兴边富民”实现“两年初见规模”目标，25个边境行政村实施集体经济项目59个，农民人均纯收入增加200多元。全市有1.8万人脱贫。

生态环境明显改善，可持续发展能力进一步增强。国家级生态示范区建设向前推进，森林覆盖率由56.66%提高到58.7%，建成沼气池2301座，城乡环境质量明显提高；率先应用国内领先的“酒精废液资源型利用技术”，实现农民增收、企业增利和环境保护共赢。土地、海洋、矿产等资源管理工作取得新进展，GEF红树林示范项目的实施得到联合国规划署充分肯定；基本农田保护和治理整顿土地市场工作通过自治区验收。坚决执行市委的决策和市人大的决议，胜利完成了集中清理整治东西湾海域任务。认真执行计划生育国策，出生人口比自治区下达指标少3602人；治理出生人口性别比偏高和开展“关爱女孩行动”试点工作取得一定成效。

精神文明、政治文明建设取得新成绩，各项社会事业全面进步。深入落实《公民道德建设实施纲要》，开展创建文明行业和诚信建设等活动，市民素质不断提高。平木村“十姐妹”等学雷锋先进群体成为精神文明建设的典范。未成年人思想道德建设进一步加强。

民主法制建设得到加强。认真执行市人大及其常委会的决议、决定，坚持向市人大常委会报告制度和政府组成人员述职制度，坚持向市政协通报制度。办理人大代表议案、建议、批评和意见94件，办复率100%；办理政协委员提案156件，办复率100%。基层民主和法制建设进一步加强，村务公开逐步规范，“四五”普法顺利推进，完成了32个一级司法所规范化建设任务。

社会稳定和安全生产工作成效明显。“严打”整治斗争深入开展，社会治安防控体系建设进一步加强，刑事案件发案率下降24%。打击走私取得新成果。打击“六合彩”等赌博活动力度加大。协助国家有关部门认真处置涉嫌撞沉法国渔船的菲律宾“西雅图商人号”船只事件，赢得有关国家的赞誉；妥善处理了多起涉外事件，维护了边境稳定。加强信访调处工作，有效地预防和处理群体性事件。安全生产得到加强，各类伤亡事故、死亡人数、受伤人数、直接经济损失四项指标均比上年下降。

双拥工作再上新台阶。积极支持部队建设，认真落实优抚政策，安置军转干部、退役士兵和随军家属395名。荣获全国、全区“双拥模范城”称号。

各项社会事业取得新成绩。推动科技成果应用和转化，开发工业新产品10个，推广农业新品种19个、先进技术13项。东兴市、港口区“普实”通过自治区验收，上思县“普九”工作顺利推进，高考本科上线率增长12.23%，创历史新高。文艺创作屡获佳绩，独弦琴《京家阿伯踩竹乐》获文化部群星金奖，是建市以来获得的最高文艺奖。防城港人民广播电台建成开播，市有线电视网络整合已完成。“非典”、禽流感、狂犬病等传染病得到有效防控；白内障复明行动和“明天计划”残疾孤儿手术康复工作顺利实施；上思疾控中心和传染病病区、东兴疾控中心建成使用，市疾控中心建设进展顺利。成功承办全国帆板冠军赛，参加全区第五届农民运动会获得好成绩。婚姻登记工作成绩显著，被民政部评为广西唯一的地级市婚姻登记先进单位。“扫黄”、“打非”、“禁盗”和网吧治理力度加大。口岸联检部门推进“大通关”，为中国—东盟博览会营造了良好的口岸环境。海上搜救工作取得新成绩。统计工作进一步加强，经济普查有序推进。质量技术监督、食品药品监管、人事人才、侨务、对台、民族、宗教、气象、档案、修志、机关事务、人民防空、国防教育、民兵预备役、老龄、妇女、儿童、残疾人、防震减灾等工作都取得新成效。

行政效能明显提高，政府自身建设得到加强。机关效能建设深入开展，全市受理效能投诉331件，办结302件，办结率91.24%。完成新一轮政府机构改革。进一步推行政企分开，组建了港口管理局和防城港务集团有限公司。政务公开有序推进，向社会公布重大决策和重大事项。市行政服务中心组建基本完成。依法行政向前推进。贯彻实施《行政许可法》，清理行政审批事项1750项，取消960项。降低行政事业性收费标准2405项，取消收费项目129项。修订了市人民政府工作规则，政府工作进一步规范。加强学习培训，政府公务员队伍素质和行政效率明显提高。深入推进廉政建设，市政府系统副处级以上领导干部签订廉政责任状，个人向市纪委递交了廉政承诺书。审计工作不断加强，在对领导干部实行离任审计、届中审计的同时，提前介入重大项目工程审计。加大了查处违纪违法案件和清理拖欠公款力度。

各位代表！

过去一年，在面临禽流感影响、运输电力紧张、旱涝等诸多困难的情况下，我们取得了显著成绩，确实来之不易。这是自治区党委、自治区人民政府和市委正确领导的结果，是全市各族人民共同奋斗的结果。在此，我谨代表市人民政府，向全市各族人民致以崇高的敬意！向驻我市的中直、区直单位，向驻军部队官兵，向所有关心、支持我市建设的各界人士和海内外朋友表示衷心的感谢！

回顾一年的工作，我们深深体会到：必须把科学发展观贯穿于经济社会发展的全过程，在加快经济发展的同时，大力发展各项社会事业，实现经济社会全面、协调、可持续发展。必须以经济建设为中心，以发展为第一要务，着力解决前进中出现的新问题，努力保持经济快速发展。必须加大招商引资力度，充分利用国际国内两种资源、两个市场，积极参与区域合作，实现招商引资新突破。必须抓重点、抓关键，大力推进事关发展全局的重大项目建设，不断增强经济发展后劲。必须坚持以人为本，切实保障人民群众的经济、政治和文化权益，促进社会和谐稳定。

在肯定成绩的同时，我们清醒地认识到，我市经济社会发展还面临不少困难和问题。主要是：经济总量小，可用财力有限；农业抵御自然灾害能力低，农村水、电、路等基础设施不够完善；电力供应紧张，疏港压力大；筹措项目建设资金压力大；历史遗留问题解决难度大；科教文卫体等社会事业还不能满足人民群众日益增长的文化生活需要；社会不稳定因素和安全生产隐患依然存在；人民群众对机关个别部门、个别工作人员的工作水平还有不够满意的地方。对这些问题，我们必须高度重视，努力加以解决。

二ＯＯ五年主要任务

今年是全面完成“十五”计划，为“十一五”计划奠定坚实基础的关键一年。根据市委的总体部署，今年市政府工作的总体要求是：以邓小平理论和“三个代表”重要思想为指导，深入贯彻党的十六大、十六届三中、四中全会精神，贯彻落实中央经济工作会议、自治区党委八届五次全会、全区经济工作会议和市委三届四次全会部署，坚持以经济建设为中心，以发展为第一要务，全面落实科学发展观，紧紧抓住当前重要战略机遇期，按照“大港口、大通道、大工业、大开放、大发展”的工作思路，更加有效地推进改革开放，加快经济结构调整，转变经济增长方式，完善港口、城市等基础设施，推进重大项目建设，大力发展临港临边工业，努力壮大县域经济，提高城乡居民生活水平，大力发展社会各项事业，切实加强精神文明和民主法制建设，着力构建和谐社会，努力实现“兴港、强市、富民”目标，加快全面建设小康社会步伐。

今年全市经济社会发展的主要预期目标是：全市生产总值增长12%左右；财政收入增长12%左右；港口货物吞吐量增长20%；工业总产值增长20%，其中国有及规模以上工业产值增长30%；全社会固定资产投资增长30%；社会消费品零售总额增长11%；外贸进出口总额增长15%，其中出口增长11%；实际利用外资2000万美元；城镇登记失业率控制在3.5%以内；城镇居民可支配收入增长6.5%；农民人均纯收入增长6%。为实现上述经济社会发展目标，今年必须更加注重港口建设；更加注重做好“三农”工作；更加注重强化工业主导地位；更加注重沿海基础设施建设；更加注重加强经济社会发展的重点领域和薄弱环节；更加注重改革开放；更加注重做好关系人民群众利益的各项工作。

一、加快港口建设步伐，全力提升港口核心竞争力

扩大港口规模和完善配套功能。以建设大型化、专业化、现代化港口为目标，加快引进战略投资者。进一步理顺港口管理体制。港务集团公司要尽快上市，增强融资能力，实现港口建设滚动发展。年内要完成20万吨级泊位及配套航道、13和14号泊位建设，加快15至17号泊位和东湾化工码头建设；开工建设5万吨级东湾航道；做好18至22号泊位、30万吨级矿石码头、30万吨级油码头、煤炭专用码头和企沙工业区配套码头等项目前期工作。争取开辟更多直航全球主要港口的航线，近期开通防城港至海参崴、高雄直航航线。突出抓好防城港发展规划和企沙半岛岸线规划，整合大、中、小港口资源，形成分工明确、各具特点的港口群。加快港口设施技术改造和工艺创新，全面提高港口装卸自动化水平。加快插排尾等村改搬迁，拓宽港区发展空间。

加快与港口相配套的路网和口岸设施建设。抓住自治区与铁道部实施铁路建设“一揽子”计划的机遇，配合做好南防铁路扩能改造；继续争取铁路部门支持，提高货物集疏运能力。东兴至江山一级公路、上思至东兴公路建成使用。完成疏港大道工程，加快上思至崇左二级公路防城港市段等项目建设。做好防城至茅岭二级公路前期工作。加大口岸建设力度，逐步完善东兴、峒中等口岸基础设施。

二、实施工业兴市战略，着力构建八大工业体系

突出抓好一批工业项目。继续实施“以港引工、以工促港、港工互动”思路，大力发展以重工业为主轴的特色产业集群，加快形成粮油加工、钢铁、电力、化工、电子、制糖、制药、特色农产品加工等八大工业体系，努力培育粮油加工、钢铁、电力、化工等四大工业支柱产业。加快新海油脂二期和大海粮油三期工程建设；力争今年五月底防城港发电厂主体工程开工建设；抓好万鑫钢铁、宏源浆纸、中油能源等项目的投产。推进大型钢铁、煤化工和大型能源等项目的前期工作。一手抓大项目的引进，一手抓中小项目的发展，努力形成产业特色突出、专业分工合理、大中小项目优势互补的工业发展格局。

加快工业区建设。按照“总体规划，分期建设”的原则和城市总体规划要求，高标准、高起点规划和建设工业区，加大投入力度，完善工业区功能。重点抓好企沙、东湾工业区规划和基础设施建设，搭建临港工业发展平台。积极推进江平工业区建设的前期工作。坚持立足当前，着眼长远，努力集聚一批项目落户工业区。

努力提高工业经济效益。认真落实我市强优工业企业奖励办法，按照“分类指导、一企一策”的要求，盘活存量资产，把工业企业做强做大。发挥大海粮油、新海油脂等骨干企业的强势效应，带动中小企业向“专、精、特、新”方向发展。加大扶持力度，抓好制糖、制药、林产等传统工业的扩建、技改和新产品开发。加强煤电油运等重要生产要素的综合协调，保障工业经济正常运行。加强协调服务，重点支持产品适销对路的企业开足马力扩大生产。抓好工业企业扭亏增盈，提高工业对财政的贡献率。

三、实施龙头企业带动战略，发展壮大县域经济

以龙头企业带动农业产业化发展。加快建立原材料基地，重点扶持海世通、扶隆香料、上思高峰集团等一批农产品加工龙头企业发展，推荐1至3家重点农业龙头企业参加自治区评选。以龙头企业促进农业结构调整，特别要改造水旱田、低产田和咸酸田，突出发展甘蔗、海水养殖、畜牧业等特色经济，在沿边公路建设一批无公害蔬菜、水果、香料等基地，力争每个区、县（市）都培育起一、二个特色优势产业。优化粮食品种结构，杂交水稻要占播种面积80%以上，粮食总产量达到24万吨，增长5%左右。

加快县域工业化进程。大力抓好港口渔洲城、防城茅岭和河西、东兴边境、上思城西等工业区建设，培育壮大上上糖业、华塑包装、仁盛制药、中天药业、鉴声电器等一批县域特色工业。通过发展工业，增强县域经济综合实力，力争年内有一个区、县（市）荣获广西县域经济发展十佳县称号。

扩大农业领域对外开放。加强与东盟各国在农业技术、动物防疫、农产品贸易等方面的合作，重点扶持扩大特色产品和有机食品的出口，集中力量建设一批高起点、高水平的农产品出口基地，吸引和鼓励国内外企业参与农业开发，使农业资源优势转化为经济优势和竞争优势。

加大扶贫工作力度。全面完成“兴边富民”三年目标和南屏瑶族乡基础设施建设大会战任务。扎实开展“兴村富民”；对首批确定的38个贫困村实行“整村推进”。加强十万山区北麓、南麓和西线贫困山区交通网络建设。努力改善少数民族聚居地生产生活条件，增进民族团结。积极推进智力扶贫和产业化扶贫，加大贴息贷款投放力度，增加贫困村农民收入。努力提高农业科技含量。加大良种良法推广力度，搞好农作物、水产养殖、畜禽良种繁育基地建设。组织实施“科技入户工程”，扶持带动能力强的科技示范户。大力普及先进农业技术，加快农业科技转化。开展扎实有效的农民转业转岗培训，年内培训农民工要达5000人以上，新增农村劳动力转移1万人以上。

大力改善农村基础设施。扎实开展全市水利基础设施建设大会战，完成除险加固病险水库29座，建成海河堤防、渠道硬化33公里，修复水利水毁工程215处，完成人饮国债项目28个。加快农村公路建设，改建等级油路70公里，开工建设乡镇客运站10个，提高通村公路比重和运输通达水平。逐步完善农村电力、通信、教育、文化、卫生等基础设施。

四、加快城市基础设施建设，逐步完善城市功能

加大城市基础设施建设力度。采取市场化运作方式，多元化筹措城市建设资金，加快市政设施建设步伐。突出抓好南北大道、东西大道等城市重点地段建设；建设拥军路东段、西湾广场东路、贵州路等市政道路，改造跨海大桥至彩虹桥、兴港大道、东兴大道等路段。重点抓好市10万吨水厂、市污水处理厂、市生活垃圾处理厂堆肥工程、城市管道燃气等基础设施建设。

大力推进城市中心区和小城镇建设。积极鼓励和支持投资者、单位和个人参与城市中心区建设，加快房地产开发，吸引民间资本投资城市中心区开发。加快市四套班子办公用房建设，确保年内搬迁办公；一批单位办公用房和职工住宅小区也要加快建设；配套完善水、电、路、通信等基础设施，带动整个中心区的建设，推进城市中心北移，加快“还岛于港”进程。利用东兴至江山一级公路、东兴至上思公路等交通干线，结合发展特色产业、旅游业和生态保护，加快推进企沙、江平、那良、峒中、在妙等重点乡镇和新村建设。

抓好城市规划和管理。搞好城市中心区的控规、重点地段的城市设计和供水、供电、通信等专项规划编制。做好企沙至东兴50公里经济带规划，促进产业合理布局。继续抓好一年一度的“迎中国—东盟博览会、美化城市环境”活动，落实烂尾楼“一帮一”责任制。抓好城市总体形象的策划、设计和包装，加大对外宣传和推介力度，进一步提高防城港市知名度，树立防城港市新形象。完善城市化政策支撑体系，着力解决户籍、社保、教育和产业取向、要素流动等问题。

五、狠抓项目落实，保持投资对经济增长的强力拉动

抓好沿海基础设施和重大项目建设。重点加快沙潭江至企沙一级公路、企沙工业区供水管网、白石牙水库、茅岭110KV输变电等10个沿海基础设施建设大会战项目建设，确保年内投入使用。平福水电厂要在上半年发电。在抓好总投资180亿元的30个在建续建项目的同时，争取开工建设防城城区防洪排涝、北仑河河堤改造、东兴污水处理厂、东兴口岸改造、企沙渔港改造、上思给水扩建及管网改造等项目。创新重大项目前期工作机制，加快推进企沙铁路支线、东湾跨海大桥、中越北仑河二桥及配套公路等项目的前期工作。落实目标责任制，认真协调解决项目建设过程中出现的困难和问题。强化项目管理，确保工程质量。

不断拓宽项目建设资金渠道。一是创新融资机制。在积极争取国家、自治区专项资金支持和政策性拨款的同时，采用BOT、TOT等项目融资方式，引导民资、外资参与我市重点基础设施项目建设，特别是支持银行与项目业主合作，建立政府、企业、银行联席会议制度，争取更多的银行贷款。积极探索激活民间资金的新路子，努力使个人存款变为投资资本。二是盘活现有政府性资产。把分散在各部门、各行业的国有资产进行有效集中，发挥政府资金的带动效应；整合土地资源，加大土地储备力度，最大限度地盘活土地资产。三是充分发挥财政资金导向作用。把项目建设资金纳入国民经济计划和财政预算，设立项目建设推进专项资金，加大对重大基础设施项目建设的支持力度。

六、全方位招商引资，提高对内对外开放水平

加大招商引资力度。一是认真落实招商引资激励机制和目标责任制，建立招商引资工作联席领导协调会议制、投资软环境监督制等新制度，动员全社会力量参与招商引资。二是认真研究招商策略，加强政府引导和服务，以企业为主体，积极推动招商引资市场化、专业化，提高招商引资成功率。三是充分利用中国—东盟博览会和中越边境商品交易会平台，采取小分队、结交友好城市等多种形式招商，着力引进浙江、江苏、广东、福建和山东等沿海地区和国外资金。四是认真跟踪落实已签订的项目，注重提高签约项目履约率、开工建设率、建成投产率。五是处理好市本级与各区、县（市）在招商引资、项目开发等方面所涉及的利益分配、资源共享问题，调动各方面招商引资的积极性。

大力发展外向型经济。全面拓展与东盟国家的经贸合作，加快发展机电、轻纺、食品等边境加工贸易和境外加工贸易，特别是利用出海出边通道优势，大力发展通道经济，努力建设出口生产、加工贸易、现代物流三大基地，提高出口产品科技含量和附加值，促进出口稳定增长。用足用活边贸政策，发展沿边经济，边境贸易成交额要增长10%以上。实施“走出去”战略，积极鼓励、引导、支持有实力的企业到越南等东盟国家投资兴业，扩大国际经济技术合作。

七、发展旅游商贸等第三产业，努力扩大消费需求

壮大特色旅游业。大力整合旅游资源，重点清理整治江山半岛和京岛金滩旅游景区。抓好西海湾景观建设。建成江山半岛旅游景区道路。规范边境旅游市场，努力把东兴市创建为广西优秀旅游城市，争创中国优秀旅游城市。采取市场运作方式，恢复防城港至越南下龙湾海上旅游航线，并采取措施确保稳定运营。加强十万大山国家森林公园景区基础设施建设，争创4A级景区。办好上思森林旅游节。加强文物保护，挖掘特色文化旅游。大力开发特色旅游商品，增加旅游收入。加大旅游宣传促销力度，拓展客源市场。全年接待游客要达168万人次，增长11%，旅游总收入要达到5.87亿元，增长10%。

搞活商贸业。做好城乡商业网点规划，合理布局城市商业中心、区域商业中心以及社区便民服务中心三级商业服务网络体系，建设一批香料、水产、木材等专业市场，促进商贸业大发展。

大力发展新兴服务业。坚决执行中央稳健的货币政策，支持银行、保险等金融服务业稳步发展。加强与国内外知名物流企业合作，大力发展现代物流业。加快社会信息化建设。规范房地产市场秩序，促进房地产业健康发展。加快发展法律、会计、评估、会展、社区服务等新兴服务业。

积极促进居民消费。落实鼓励消费的政策措施，培育住房、汽车、高档电器、信息服务等消费热点，促进消费结构升级。积极发展消费信贷，促进个人信用消费。深入整顿和规范市场经济秩序，重点抓好直接关系人民群众身体健康和生命安全的食品、药品等专项整治，创造良好的消费环境。

八、全面推进各项改革，建立健全可持续发展的制度保障

推进企业国有产权和国有资产管理体制改革。一要年内完成企业国有产权改革工作任务。抓住制约企业改革的难点和重点，寻求对策、探索路子，多形式、多渠道加以重点突破；设立企业改革资金专户，为企业改制提供资金支撑；严格企业国有产权改革程序，依法规范运作，防止暗箱操作；处理好改革与稳定的关系，做好企业职工的安置和分流。二要建立健全国有资产监督管理体制，防止国有资产流失，维护出资人和职工的合法权益。

推进农村各项改革。深化农村税费改革，建立健全防止农民负担反弹的长效监管机制。依法稳定农村土地承包关系。深化农村土地流转制度改革，实行最严格的耕地保护制度，加强农民宅基地管理。抓好国有水利体制改革试点，推进以组建农民用水合作组织为主要形式的小型水利管理模式。加快国有粮食购销企业转变经营机制和农村信用社改革。积极稳妥地推进乡镇机构和县乡公共财政体制改革，扎实做好乡镇撤并工作。

推进财税体制改革。围绕构建公共财政体制，继续推进部门预算改革，提高预算透明度，增强预算约束力。稳步推进财政国库管理制度改革试点工作。进一步完善“收支两条线”管理制度。全面推行管采分离，扩大政府采购规模和范围，进一步规范政府采购行为。坚决执行中央稳健的财政政策，大力培育财源，依法加强收入征管，合理安排支出。

推进投资体制改革。简化项目投资审批程序，扩大社会资本投资领域，放宽市场准入条件，确立企业投资主体地位，规范政府投资行为。建立政府投资责任追究制度，实行政府投资项目公示制和重大投资项目后评估制度。规范投资项目决策和工程咨询、设计、施工、监理等工作，提高项目建设效益和质量。

大力发展非公有制经济。毫不动摇地鼓励、支持和引导非公有制经济发展，切实保护私有财产权，确保非公有制企业与其它企业享受同等待遇。加强政府服务、管理和监督，把非公有制经济发展纳入经济发展总体规划。运用担保、贴息等手段，拓宽非公有制企业融资渠道，推动非公有制企业体制、机制、技术和管理创新，引导和扶持一批非公有制经济龙头企业做大做强。

九、坚持以人为本，构建文明和谐社会

抓好就业再就业和社会保障工作。把就业工作列入经济社会发展目标，新增城镇就业9500人，城镇下岗失业人员实现再就业1200人。继续巩固“两个确保”，力争参加养老、失业、医疗、工伤和生育保险人数达到18万人以上。继续抓好“一个低保”，逐步完善社会弱势群体救助帮扶长效机制。加大五保村建设力度，完善农村五保户和特困家庭生活保障制度。做好救灾救济工作。

大力发展科教文卫体等社会事业。构建“农村科技信息服务网”，实现全市乡镇科技信息网络“乡乡通”；加强与知名大学和科研院所的科技合作交流，推进科研成果的转化利用。加大“两基”攻坚力度，确保年内上思县“普九”达标；抓好“义教”、“危改”和农村寄宿制学校建设，基本消除D级危房；启动高等职业技术学院建设，大力发展职业技术教育；强化师资队伍建设，发展民族教育。挖掘民族文化文物，争取京族文化列入全国民族民间文化保护工程试点项目；做好市文化艺术中心和市公共图书馆的筹建工作。实施“村村通”工程，提高广播电视覆盖率；抓好广播电视传输网络升级改造，逐步推进广播电视数字化。建立健全突发公共卫生事件应急机制和城乡困难群众大病救助机制；抓好市第一人民医院传染病病区、市紧急医疗救援中心国债项目建设；落实防治“非典”、禽流感、狂犬病、艾滋病等传染病的各项措施；加强计划免疫工作；推进农村初级保健和东兴市新型农村合作医疗试点。推动全民健身，提高体育运动竞技水平；争取国家体育总局在我市建设水上训练基地；加快市体育馆前期工作。

加强人力资源开发。牢固树立人才资源是第一资源的观念，着力抓好培养、吸引、用好人才三个环节。强化人才培训，注重实践锻炼，有计划地选派人才到广东、浙江、江苏等发达地区挂职。完善人才市场体系，促进人才合理流动。深化人事制度改革，建立和营造鼓励优秀人才脱颖而出、人尽其才的有效机制和良好环境。

推进人口、环保、国土等工作。加强人口与计划生育工作，确保计划生育各项指标控制在自治区下达的计划内；按照属地管理原则，加强区直农林场和城区流动人口计划生育管理；综合治理出生人口性别比偏高问题，对农村部分计划生育家庭实行奖励扶助。全面推进国家级生态示范区建设；推广制糖业“酒精废液资源型利用技术”，大力发展循环经济；加快造林绿化和退耕还林步伐，加强珠江上游十万大山水源林保护，强化防城江、明江和北仑河的水污染防治。认真抓好国土资源管理体制改革，统筹安排好各类建设用地用海；打击非法淘矿采砂等行为，加大海洋执法监察力度，巩固发展东西湾海域清理整治成果。认真做好全市经济社会发展“十一五”规划和有关专项规划编制。加强统计特别是经济普查工作。关心和支持老龄、妇女、儿童、残疾人工作。依法管理宗教事务。抓好对台和侨务工作。审计、外事、气象、档案、修志、新闻出版、机关事务、防震减灾等各项工作都要取得新的成绩。

全力维护社会稳定，努力确保安全生产。进一步加强社会治安防控体系建设，努力建设“平安防城港”、“和谐防城港”、“稳定防城港”。严密防范和坚决打击境内外敌对势力和“法轮功”等邪教组织的渗透、分裂和破坏活动。严厉打击暴力、走私、毒品等严重刑事犯罪，力争刑事案件下降10%以上。深入打击“六合彩”等赌博违法犯罪行为，维护正常的社会秩序。健全正确处理人民内部矛盾的工作机制，认真做好人民群众来访来信工作，积极预防和妥善处置群体性事件。努力完善安全生产监督管理体制和机制，认真落实安全生产责任制和责任追究制，突出抓好危险化学品、矿山、交通运输、民爆物品、烟花爆竹、公众聚集场所等安全生产专项整治工作，积极防范各类事故发生，确保人民群众生命财产安全。

十、加强精神文明和政治文明建设，为经济社会发展提供精神动力和法制保障

推进民主法制建设。自觉接受市人大及其常委会的法律监督和工作监督，坚持向市人大及其常委会报告制度和述职制度，坚决执行市人大及其常委会的决议、决定，认真办理人大代表议案、建议、批评和意见。坚持向市政协通报制度，加强与市政协的联系，自觉接受市政协和各民主党派、工商联、无党派爱国人士的民主监督，认真办理政协委员提案。充分发挥工会、共青团、妇联等群团组织的桥梁纽带作用。巩固和扩大基层民主，完善村民自治、社区民主管理和企业管理制度，推进村务公开和厂务公开。加强人民调解组织建设。深入开展“法律进万家”活动，确保“四五”普法通过自治区验收。加强法律援助工作，完善法律服务体系。

加强精神文明建设。进一步贯彻《公民道德建设实施纲要》，加强职业道德、社会公德、家庭美德教育。进一步实施国门形象创建工程，大力推进“边海文明示范带”和群众性精神文明创建活动。广泛开展社区和广场文化活动，不断提高市民素质和城市文明程度。积极引导市民移风易俗。继续开展诚信教育，努力构建社会信用体系。加强和改进未成年人思想道德建设。唱响团结、稳定、鼓劲、发展主旋律，营造良好的舆论环境。

大力支持国防建设。深入开展拥军优属、拥政爱民活动，积极为驻军做好服务和保障，妥善安置军队转业干部、退役军人和随军家属，增进军政军民团结，努力提高双拥工作水平。加强全民国防教育，推进国防动员、人民防空、交通战备和民兵预备役建设。

全面推进服务型政府建设

新的一年，我们要按照科学执政、民主执政、依法执政的要求，坚持全心全意为人民服务的宗旨，以建设“规范、高效、廉政、务实、民主、团结”的现代服务型政府为目标，以保持共产党员先进性教育活动为动力，以机关效能建设为载体，不断提高依法行政能力和服务、管理水平。

建设规范政府。全面推进依法行政，认真落实《行政许可法》和《全面推进依法行政实施纲要》，善于运用法律手段组织、管理经济和社会事务。紧密结合实际，加强制度建设。健全行政执法责任制，强化监督约束机制，消除以权代法、以言代法现象。加快政府职能转变，更好地履行社会管理和公共服务职能。进一步深化行政审批制度改革，依法规范行政机关办事程序和办事时限，进一步健全工作报告制度和强化工作督查制度，促进政府工作的规范化、制度化和法制化。

建设高效政府。加强机关效能建设，把有利于机关作风转变和工作效率提高的各项工作制度真正落到实处。提倡效能革命，破除各种不利于政府工作提速的思想观念、行为模式和惯性机制，加快管理创新，切实提高政府服务质量和行政效率。今年三月底前市行政服务中心要正式运作，实行一个“窗口”服务，方便投资者和群众办事。推行电子政务，整合信息网络资源，开通市政府公众信息网，提高政务处理信息化、网络化水平。实施“公务员素质提升工程”，全面提高综合素质，不断增强办实事、解难题、创一流的能力。着力建设办事程序少、速度快、成本低、水平高、群众满意的高效政府。

建设廉政政府。全面贯彻中央纪委第五次全会和国务院第三次廉政工作会议精神，认真实施《建立健全教育制度监督并重的惩治和预防腐败体系实施纲要》，进一步落实党风廉政建设责任制，切实做到清正廉洁、用权为公、执政为民。坚持标本兼治、综合治理、惩防并举、注重预防的方针，扎实推进政府系统廉政建设和反腐败工作。进一步加强监察、审计工作，严肃查处违纪违法案件。各级机关单位都要自觉接受审计监督，对存在问题要认真整改。既要坚决治腐，也要从严治庸，决不允许行政不作为、乱作为。

建设务实政府。各级政府及其工作人员特别是领导干部都要大力弘扬求真务实精神，树正确政绩观，走实干从政路，把实现好、维护好、发展好广大人民群众的根本利益作为工作的出发点和落脚点，努力削“文山”、填“会海”、减应酬，拿出更多的时间和精力，深入基层，深入群众，深入实际，察真情、知民意、办实事。不搞形式主义，不摆花架子，不做表面文章，始终做到重实绩、说实话、干实事、求实效。对弄虚作假、欺上瞒下的行为要坚决查处！

建设民主政府。按照民主执政的要求，自觉接受社会监督。全面推行政务公开，实行社会公示和社会听证制度，建立政府新闻发言人和新闻发布会制度，增强政府运作的透明度和公众参与度，保障人民群众依法享有更多的知情权、参与权和监督权。要健全重大问题集体决策制度和专家咨询制度，广泛听取各方面的意见和建议，实现决策科学化、民主化。

建设团结政府。各级政府和政府各部门都要增强大局观念和责任意识，坚决执行市委、市政府的决策，团结一致，上下一心，聚精会神搞建设，一心一意谋发展。坚决维护市委、市政府的权威，严明纪律，令行禁止，绝不允许当面一套，背后一套。要充分调动和切实保护广大干部群众投身改革发展的积极性、主动性和创造性，努力形成加快发展的强大合力。

各位代表!

新的一年，面对新形势、新任务、新要求，我们深感责任重大、使命光荣！人民群众对政府的信任和支持，给了我们无穷的力量和必胜的信心！我们一定要紧密团结在以胡锦涛同志为总书记的党中央周围，高举邓小平理论和“三个代表”重要思想伟大旗帜，全面落实科学发展观，在市委的领导下，紧紧依靠全市各族人民，坚定信心，迎难而上，开拓创新，扎实工作，在“兴港、强市、富民”和全面建设小康社会的伟大征程中，迈出新的步伐！
